# Unveiling the Potential of Lactic Acid Bacteria from Serbian Goat Cheese

**DOI:** 10.3390/foods13132065

**Published:** 2024-06-28

**Authors:** Mirjana Ž. Grujović, Katarina G. Marković, Susana Morais, Teresa Semedo-Lemsaddek

**Affiliations:** 1 Department of Science, Institute for Information Technologies, University of Kragujevac, Jovana Cvijića bb, 34 000 Kragujevac, Serbia; 2 CIISA—Center for Interdisciplinary Research in Animal Health, Faculty of Veterinary Medicine, University of Lisbon, 1300-477 Lisbon, Portugal; 3 Associate Laboratory for Animal and Veterinary Sciences (AL4AnimalS), 1300-477 Lisbon, Portugal; 4 BioISI—Biosystems & Integrative Sciences Institute, Faculty of Sciences, University of Lisbon, 1749-016 Lisbon, Portugal

**Keywords:** goat cheese, lactic acid bacteria, safety assessment, technological features

## Abstract

This study aimed to unleash the potential of indigenous lactic acid bacteria (LAB) originating from traditionally made Serbian goat cheese. Following the isolation and identification of the LAB, the safety aspects of the isolates were evaluated through tests for hemolytic activity and antibiotic sensitivity. The selected isolates were then tested for various technological properties, including growth in methylene blue, proteolytic activity, acidification, curd formation ability in both pure and enriched goat milk, diacetyl production, antagonistic potential against other LAB, and biofilm formation ability. The results indicated that *Lactococcus* spp., *Lacticaseibacillus* spp., and *Lactiplantibacillus* spp. did not exhibit α or β hemolysis, while enterococci displayed α hemolysis. A higher number of isolates demonstrated sensitivity to ampicillin, tetracycline, and streptomycin, while sensitivity to gentamicin and vancomycin was strain-dependent. Based on the evaluation of technological properties, *Lacticaseibacillus paracasei* M-1 and *Lactiplantibacillus plantarum* C7-7, C7-8, and C14-5 showed promising characteristics. Additionally, *Lactococcus lactis* subsp. *lactis* strains C0-14 and C21-8 emerged as promising candidates with notable technological properties. Notably, certain indigenous strains LAB exhibit promising technological properties and safety profiles. These characteristics make them suitable candidates for use as starter or adjunct cultures in goat’s milk cheese production, potentially enhancing the quality and safety of the cheese as well as hygiene practices among small-scale dairy producers.

## 1. Introduction

Goat milk is widely used for an array of artisanal and commercial products, such as goat cheese, which are appreciated for their rich taste and texture, as well as nutritional value [1]. Foods derived from goat milk are known to contain unique flavors and aromas, alongside health benefits like superior digestibility in comparison with cow milk, mainly due to its lower lactose content. These products are also rich in nutrients such as calcium, proteins, and certain vitamins [2]. Nonetheless, it is important to highlight that goat milk composition may vary, depending on numerous factors such as animal breed, season, and the animal’s diet [2,3,4,5].

The Republic of Serbia boasts a rich tradition of dairy products, reflecting its cultural heritage and the wisdom of generations of producers [6,7,8] Among the most popular products are white-brined cheeses and kajmak, commonly sold at open markets, which serve as vibrant hubs for preserving culinary traditions and fostering community interactions [6,8].Traditional cheese production in Serbia predominantly occurs in small-scale dairies using raw or pasteurized milk. This traditional process does not use starter cultures but relies on natural microbiota and rennet for coagulation [9,10]. Cheeses made from raw milk can present several issues, such as the potential presence of pathogenic microorganisms like *Listeria monocytogenes*, *Salmonella* spp., *Escherichia coli*, and *Staphylococcus aureus*, which pose significant health risks [6,11]. Additionally, while yeasts and molds are essential for producing certain cheese varieties, their uncontrolled growth can adversely affect both food quality and food safety [12]. The uncontrolled fermentation process can lead to variability in flavor, texture, and overall quality [6,10]. These findings highlight the need for improved manufacturing practices and milking operations [11]. Collectively, the data emphasize the necessity for better food safety and hygiene practices among small-scale dairy producers. Enhancing these practices is crucial for reducing microbial contamination and improving both the quality and safety of traditionally made dairy products in the Serbian market [6]. To mitigate these issues, the utilization of starter cultures containing autochthonous LAB is recommended [13]. Autochthonous LAB are well-adapted to the local environment and can outcompete pathogenic bacteria, ensuring microbiological safety and contributing to consistent quality and flavor development [14]. Additionally, these LAB enhance the sensory attributes of the cheese, reflecting unique regional characteristics, and produce antimicrobial compounds that inhibit spoilage organisms, thereby extending the shelf life of the cheese [15,16].

Raw milk cheeses exhibit a particularly rich microbiota compared to pasteurized milk cheeses, harboring mainly lactic acid bacteria (LAB), which are a diverse group of microorganisms that play a vital role in the production and preservation of dairy products, including goat cheese [16]. In fact, traditional cheeses are known to comprise various LAB genera, namely *Lactococcus*, *Lactobacillus,* and *Enterococcus*, which are generally the dominant microbial groups present in these dairy products [7]. During milk fermentation, LAB convert lactose into lactic acid, a process that contributes to the characteristic tangy taste and creamy texture of goat cheese. Additionally, proteolysis and lipolysis, occurring during the ripening stage, are also crucial in taste development. Moreover, these bacteria produce a plethora of metabolites, including flavor compounds, enzymes, and antimicrobial agents, all of which influence cheese development and maturation [2,16]. In recent years, there has been a growing interest in research focused on isolating and characterizing LAB from goat milk and cheese. These efforts seek to identify indigenous LAB with desirable traits such as rapid acidification ability, robustness against environmental stressors, and ability to produce unique flavor compounds [17]. Despite the pivotal role of LAB in goat milk and cheese production, various factors such as milk composition, processing techniques, and environmental conditions influence the dynamics and functionality of these bacteria [18,19]. Hence, understanding the interactions between LAB and goat milk constituents would be beneficial for optimizing cheese-making processes and ensuring product quality and safety to meet consumer expectations and safety regulations.

LAB involved in cheese production include both starter lactic acid bacteria (SLAB) and non-starter lactic acid bacteria (NSLAB). SLAB initiate the fermentation process and may originate naturally from the milk microbiota (autochthonous microorganisms) or be intentionally selected and added as adjunct cultures to ensure better control of the fermentation conditions. The utilization of LAB as starter cultures offers several advantages in goat cheese production, allowing cheesemakers to exert greater control over the fermentation process, which could result in consistent product quality and sensory properties. Overall, SLAB plays an important role in milk acidification as well as in flavor development [20]. Moreover, starter cultures have the potential to enhance the safety and shelf-life of goat cheese by outcompeting spoilage microorganisms and pathogens through the production of lactic acid (and consequent acidification of the medium) and bacteriocins, thereby ensuring product stability and reducing the risk of foodborne illnesses [16]. On the other hand, NSLAB, originating from the milk and cheesemaking facilities, are essential during the ripening stages, contributing to cheese quality and flavor [10,16]. Moreover, NSLAB isolated from cheeses can have interesting technological and probiotic properties, making them putative candidates for additional future use as starters in other fermentation processes, due to specific characteristics like antimicrobial activity or resistance to suboptimal conditions. Briefly, microorganisms from this bacterial group produce a wide range of compounds, such as dyacetil, acetoine, hydrogen peroxide, bacteriocins, volatile compounds, and bioactive peptides. These metabolites exhibit antibacterial and antifungal activity, highlighting the growing interest in their antagonistic potential against food spoilage and pathogenic microorganisms [7,20,21,22,23]. Among these molecules, bacteriocins are particularly relevant, as they have a role in the management of spoilage and potentially pathogenic bacteria like *Listeria monocytogenes*, *Escherichia coli*, or *Salmonella* spp., without harming beneficial bacteria or altering the organoleptic features of the final product. These traits make LAB suitable for use as natural preservatives in food products to extend shelf life and contribute to product safety [24,25]. It is also noteworthy to mention that some LAB strains are Exopolysaccharide (EPS) producers. EPSs are carbohydrates secreted by bacteria that have the potential to offer a wide array of health and industrial benefits such as protecting bacteria from environmental stressors, helping in cells’ adhesion to surfaces, and being involved in biofilm formation facilitating the colonization of various environments [21].

Overall, LAB utilization in goat cheese production aligns with the consumer demand for natural and minimally processed foods [26]. As aforementioned, LAB-driven fermentation not only enhances the sensory attributes of goat cheese but also offers potential health benefits, including improved digestibility and bioavailability of nutrients, along with enrichment of the final product with probiotic properties [20,27]. Nonetheless, to be considered safe for usage in food production, microorganisms must be included in an official list of approved microbes according to a series of pre-defined criteria. These lists are the Generally Recognized as Safe (GRAS) list in the United States and the Qualified Presumption of Safety (QPS) list in Europe [16]. Among the most commonly isolated bacteria from cheese, members of the former genus *Lactobacillus* stand out because of their long history of safe use in a variety of foods. On the contrary, genera such as *Enterococcus* have not yet obtained the aforementioned safety status [20], being dependent on a case-by-case evaluation.

Among the abovementioned safety criteria, antibiotic resistance is an increasing concern, due to potential horizontal gene transfer between bacteria naturally present in food and foodborne pathogens sharing the same ecological niche [28,29]. LAB from food products may potentially harbor resistance genes due to exposure to antibiotics in various environments. The use of antibiotics in veterinary medicine and agriculture may contribute to the selection of resistant strains in animals used for food production, and these resistant bacteria (or associated genetic determinants) may be transmitted to humans along the food chain [30], turning essential in the evaluation of antibiotic resistance in food-related microbes [28,29].

On the other hand, it is also very important to evaluate biogenic amine production, which may pose a concern due to their potential health risks when present in high concentrations [31]. Cheese is the food product in which these substances reach higher concentrations, depending on ripening time and the type of microorganisms involved in the fermentation process due to the breakdown of proteins during cheese ripening. Controlling the levels of biogenic amines in the final product is therefore crucial to ensure food safety [32,33].

Another relevant feature associated with food microbiota is the ability for biofilm production, either in single or mixed cultures [34]. In food science, biofilms can be associated with a dual role, being considered either detrimental or beneficial. Briefly, putative foodborne pathogens in biofilm state present increased resistance to antimicrobials or disinfectants, which contribute to their persistence on food production surfaces and may lead to cross-contamination or horizontal gene transfer events [35]. On the contrary, biofilms containing technological microbes are known to positively impact the quality, biochemical composition, and sensory properties of the final product [36]. Given LAB’s influence on cheese maturation, the ability to form biofilms may allow these microorganisms to endure on surfaces used for cheese production, as well as in the cheese itself, and these strains may colonize additional cheese batches once they are not removed by regular cleaning processes, lengthening product standardization time [37].

Nonetheless, despite the widespread use of LAB in dairy fermentation, there is still limited knowledge about the main features associated with Serbian goat cheese microbiota and implications for product quality and safety. By elucidating the functional properties, technological aspects, and safety properties of indigenous LAB strains, it may be possible to identify new opportunities for innovation and quality improvement. Thus, the aim of this study is to explore the potential of LAB isolated from Serbian goat cheese in terms of microbial diversity, safety, technological features, and biofilm-forming ability.

## 2. Materials and Methods

### 2.1. Cheese Manufacture and Sampling

The cheese under investigation originated from artisanal production within a rural setting in the Pajsijević village of Central Serbia during the spring of 2021, due to the most favorable microclimatic conditions for cheese ripening. Freshly hand-milked goat milk, characterized by a pH of 6.6, was meticulously collected post-morning and evening milking sessions and then promptly stored in a cellar. Cellar conditions are crucial in traditional cheese production, providing a controlled environment that supports the development of specific flavors, textures, and overall quality. Key factors include maintaining a stable temperature between 10 and 15 °C, managing humidity levels between 80 and 90%, and ensuring good air circulation. The utensils used in the cellar are made from materials like wood and stone. Upon collection, the raw milk underwent filtration through a gauze into an enameled receptacle and was subsequently heated to a precise temperature of 32 °C. Liquid rennet (Sirela, consisting of 85% chymosin and 15% pepsin) was added at a ratio of 28 mL per 10 L of milk, with continuous agitation. Notably, no bacterial starter cultures were used in the cheese production process. Following a period of 30–60 min, whey separation occurred, and the resultant coagulated mass was methodically segmented into cubes using a knife. Further manipulation involved the agitation of the cheese mass using a wooden implement, facilitating the redistribution of components within the mixture. Subsequently, the cheese was draped with gauze and left undisturbed for a brief period of 15 min before being transferred to fresh gauze material. An overnight resting period ensued to facilitate whey drainage. Salt application, at a rate of 10 g per layer of curd, was administered the following morning, and subsequent cubing of the cheese mass occurred. These cubes, weighing about 70–80 g, were then submerged in a brine solution (prepared by salting whey at a ratio of 1 L per 50 g of salt) and layered onto wooden boards. A covering of cotton cloth, topped with a wooden board and a 1 kg marble stone, ensured appropriate pressure and environmental conditions conducive to ripening. This aging process unfolded within a cellar environment maintained at temperatures ranging from 15 to 16 °C, spanning a duration of 28 days.

For the present study, three cubes of cheese samples (200 g) were collected from the container immediately after manufacturing, i.e., immediately before storage (day 0), and during the subsequent 28 days (i.e., on the 7th, 14th, 21st, and 28th days of ripening) from the same batch. These samples were aseptically transported to the microbiology laboratory and maintained at 4 °C until processing. Analysis occurred within 24 h of sampling. Additionally, raw goat milk was also investigated.

### 2.2. Microbial Isolation and Identification

For the isolation, characterization, and identification of LAB in goat cheese, a composed sample (200 g) was taken with a sterile spoon, placed in a sterile container, and 10 g of the working sample was aseptically measured. This working sample was homogenized in 90 mL of 2% sodium citrate solution (pH 7.5) previously heated at 45 °C and thoroughly mixed in a vortex until complete homogenization was achieved. Successive 10-fold dilutions with 2% sodium citrate (up to 10^−7^) were then prepared.

For microbiological analysis of goat milk, undiluted fresh goat milk was used. Subsequently, 1 mL of each cheese sample dilution and 1 mL of fresh goat milk were poured into Petri dishes and mixed with selective media: MRS agar pH 6.5 (Torlak, Belgrade, Serbia) was used for presumptive lactobacilli, M17 agar pH 7.2 (Merck, GmbH, Darmstadt, Germany) for presumptive lactococci, and bile esculin agar pH 7.1 (BEA, Torlak, Belgrade, Serbia) for presumptive enterococci, following the method described by Mannu et al. [38] but with modifications. After solidification, M17 and MRS agar were covered with a thin layer of the same medium to establish microaerophilic conditions. After an incubation period of 72 h at 32 °C, plates containing between 30 and 300 colonies were selected for enumeration. The number of bacteria was expressed in colony-forming units (CFU)/g of cheese. Preparation of test samples, initial suspensions, and decimal dilutions were carried out according to ISO 6887-5:2020 [39].

Single colonies were randomly picked from M17, MRS, and BEA agar plates, which were used for enumerating bacterial colonies, and streaked onto new agar plates for purification. All isolates underwent microscopic observations, Gram staining, and catalase tests. Furthermore, Gram-positive and catalase-negative LAB isolates were identified to the genus level using tests described in Muruzović, Mladenović, Žugić-Petrović and Čomić [7] and Muruzović, Mladenović, Djilas, Stefanović and Čomić [9], including growth at pH 3.5, 4.0, 6.5, and 7.0 in modified MRS and M17 broth, growth at 15 and 45 °C in MRS and M17 broth, growth at 4.0, 6.5, and 8.0% (wt/v) NaCl in MRS and M17 broth, production of carbon dioxide from glucose by subculturing the isolates in MRS broth with Durham’s tubes, growth and production of slime on MRS agar with sucrose (20.0 g/L), L-arginine, Esculin, and Hippurate hydrolysis, and citrate utilization [40,41,42,43].

Isolates from the former genera *Lactobacillus* and *Lactococcus* were aerobically incubated at 37 °C for 48 h and identified to the species level using API 50CH tests (BioMérieux, Montalien-Vercien, France) and the API website. Enterococci were identified using Microgen Strep ID (Microgen Bioproducts, (Camberley (Surrey), United Kingdom) according to the manufacturer’s procedure.

All isolates were stored at −20 °C and −80 °C in M17 (for cocci) and MRS (for rods) broth containing 20% glycerol (*v*/*v*) [38]. Working cultures were revitalized by two consecutive transfers in M17 or MRS broth at 37 °C.

Final microbial identification was conducted using MALDI-TOF mass spectrometry, as described in detail by Muruzović, Mladenović, Djilas, Stefanović and Čomić [9]. Briefly, samples were prepared using a standard protein extraction method, with some modifications. Overnight cultures (500 μL) in MRS broth (for rods) or M17 broth (for cocci) were centrifuged at 12,000 rpm for 5 min at 4 °C. After discarding the supernatant, the pellet was resuspended in 300 μL of distilled water and 900 μL of absolute ethanol. Following vortexing and centrifugation at 13,000 rpm for 2 min at 4 °C, the supernatant was removed and the pellet was dried at 55 °C for at least 30 min. Subsequently, 50 μL of 70% formic acid was added and mixed thoroughly, and then 50 μL of 50% acetonitrile was added. After another centrifugation at 13,000 rpm for 2 min at 4 °C, 1 μL of supernatant was placed on a 96-spot MALDI target plate. The plate was allowed to dry for 10 min before overlaying with 1 μL of the matrix solution (Bruker Matrix HCCA; α-Cyano-4-hydroxycinnamic acid). Each culture was measured once and the results were expressed as MALDI-bioTyper matching scores (ranging from 0.000 to 3.000), with values ≥2.00 considered correct identifications at the species level.

### 2.3. Safety Evaluation

The safety aspect of the test involved examining the hemolytic activity and resistance of the bacteria to selected antibiotics.

#### 2.3.1. Hemolytic Activity

Isolate’s ability to synthesize extracellular proteins, specifically hemolysins, on blood agar plates was investigated [44]. Hemolytic activity was tested on sheep blood agar plates incubated at 37 °C for 24 h. *S. aureus* ATCC 25923 was used for quality control. The β-hemolytic reaction leads to complete lysis of erythrocyte cells, resulting in a clear halo around the colony, while the α-hemolytic reaction involves the appearance of a greenish color. A γ-hemolytic reaction indicates that the strain showed no hemolytic activity.

#### 2.3.2. Resistance to Antibiotics

LAB antibiotic susceptibility was investigated using the microdilution method with resazurin, and the minimum inhibitory concentration (MIC) was determined [45]. Ampicillin, tetracycline, gentamicin, streptomycin, and vancomycin (Sigma Chemicals Co., USA) were used in concentrations ranging from 0.05 to 4000 µg/mL for this study. Twofold serial dilutions of the antibiotics were prepared in sterile 96-well microtiter plates containing 0.1 mL of MRS broth (Torlak, Belgrade, Serbia) per well for rods and 0.1 mL of M17 broth (Merck, GmbH, Darmstadt, Germany) per well for cocci. The microtiter plates were inoculated with suspensions to achieve a final concentration of 5 × 10^5^ CFU/mL. Bacterial growth was monitored by adding resazurin (Alfa Aesar GmbH & Co., Karlsruhe, Germany), a blue nonfluorescent dye that turns pink and fluorescent when reduced to resorufin by oxidoreductases in viable cells. The inoculated microtiter plates were incubated at 32 °C for 24 h. The MIC was defined as the lowest concentration of the antibiotics that prevented the resazurin color change from blue to pink. The method was described in detail by Muruzović et al. [46].

### 2.4. Technological Features

Technological features were evaluated through tests including growth in the presence of methylene blue, proteolytic activity, assessment of acidification and curd formation ability in both pure and enriched goat milk, diacetyl production as well as detection of LAB antagonistic potential, and evaluation of its biofilm formation ability.

#### 2.4.1. Growth in the Presence of Methylene Blue

The ability of the isolates to grow in pasteurized milk containing 0.1% methylene blue was tested by inoculating the milk with a 2% (*v*/*v*) bacterial inoculum. Incubation was carried out at 37 °C for 24 h. After incubation, the color change was observed to determine the bacterial capacity to reduce methylene blue. Pasteurized milk containing 0.1% methylene blue, inoculated with *L. plantarum* LP 299v, served as a positive control. A negative control consisting of non-inoculated milk with methylene blue was also included.

#### 2.4.2. Proteolytic Activity of Tested LAB

The proteolytic activity was examined according to Harrigan and McCance [47], with slight modifications. Briefly, the proteolytic activity of tested LAB was evaluated on a medium composed of nutrient agar and milk (1.6% milk fat) in a 1:1 ratio. Bacteria were aseptically transferred onto the medium and allowed to incubate at 37 °C for 24 h. The appearance of a transparent zone around bacterial colonies indicated proteolytic activity. *Bacillus subtilis* ATCC 6633 served as a positive control, while *Escherichia coli* ATCC 25922 was used as a negative control.

#### 2.4.3. Milk Acidification and Curd Formation

Milk acidification ability was assessed by measuring pH and curd formation in both pure goat milk and enriched goat milk. Enriched goat milk was prepared by adding 2% glucose and 1% yeast extract, followed by gentle heating until complete dissolution. In such prepared milk, a bacterial inoculum (2% (*v*/*v*)) was added. pH measurements were taken after 6 and 24 h of incubation at 32 °C using a pH meter (Basic pH meter, Arvada, CO, USA). Additionally, the appearance of curd and gas in both types of milk was monitored. Negative controls consisted of non-inoculated milk and enriched milk [40,41,42,43]. The pH values are presented as the means of the results obtained from all isolates within the same genus.

#### 2.4.4. Diacetyl Production

Diacetyl production was tested as follows: LAB were inoculated in reconstituted skimmed milk for 16 h. To 1 mL of coagulated milk, 0.1 g of creatinine and 0.1 mL of 30% NaOH (by mass per volume) were added. Diacetyl generation was indicated by the formation of a red ring at the top of the tubes after 2 h.

#### 2.4.5. Biogenic Amine Production

The production of tyramine was assessed using medium containing the following components: Peptic digest of animal tissue (5 g/L), beef extract (5 g/L), dextrose (0.50 g/L), bromocresol purple (0.01 g/L), cresol red (0.005 g/L), pyridoxal (0.005 g/L), pH 5.3, supplemented with histidine and tyrosine at 0.5% of final concentration. A bacterial suspension (10^9^ CFU/mL) was made from a plate culture in a decarboxylase medium without amino acids and incubated for 2–5 days at 30 °C. Subsequently, 0.2 mL of pre-grown culture was inoculated into 3 mL of modified medium, with or without the amino acid. Following anaerobic incubation at 37 °C for 1–7 days, observations of color variations in the broth culture from yellow to purple confirmed positive reactions [48].

#### 2.4.6. Detection of Antagonistic Potential

The antagonistic potential of LAB members was screened using the agar-well diffusion method [49]. Three standard strains, *Escherichia coli* ATCC 25922, *Proteus mirabilis* ATCC 12453, and *Staphylococcus aureus* ATCC 25923, were employed. *Escherichia coli* G14 [10] and *Klebsiella pneumoniae* (a human isolate, generously provided by the Institute of Public Health Kragujevac) were used as indicator strains. The microbial collection was maintained in a 20% glycerol/medium mixture at −80 °C. Before use, indicator bacteria were revitalized by two consecutive transfers in nutrient agar (Torlak, Belgrade, Serbia) at 37 °C.

Soft nutrient agar (0.7%, *w*/*v*), containing the indicator strains, was overlaid onto MRS plates. Wells were created in the lawn of hardened soft agars. 100 μL aliquots of LAB overnight cultures (18 h) were centrifuged at 10000 rpm for 30 min at 4 °C, adjusted to pH 6.5 by adding 12 M NaOH, and filter sterilized. The neutralized and filtered supernatant was placed in the wells (6 mm) and assayed for antagonistic activity against indicator bacteria. The plates were then incubated overnight at 37 °C. A clear zone of inhibition around the well was measured, and the size of the well was subtracted from the total zone diameter to compensate for the background zone.

For comparison, the sensitivity of indicator strains to the following antibiotics was tested in parallel: chloramphenicol (30 µg), Amoxicillin (25 µg), and tetracycline (30 µg). The zones of inhibition were interpreted according to EUCAST guidelines (2024).

#### 2.4.7. Biofilm Formation Assay and Quantification

Indigenous LAB were assessed for their ability to form biofilms following protocols described by O’Toole et al. [50] and Stepanović et al. [51], with some modifications shown in detail in Grujović et al. [52]. Tissue culture 96-well microtiter plates (Sarstedt, Germany) were filled with 100 μL of MRS broth in each well, and 50 µL of fresh bacterial suspension containing approximately 10^8^ CFU/mL was added to each well. The plates were then incubated at 37 °C for 48 h. After incubation, the contents of each well were gently removed and the wells were washed with 200 µL of sterile 0.85% saline to remove free-floating bacteria. The biofilms formed by adherent cells were fixed with 100 μL of methanol and stained with 0.1% (*w*/*v*) crystal violet, followed by incubation at room temperature for 20 min. Excess stain was rinsed off by washing with deionized water and solubilized with 200 mL of 96% ethanol. The optical densities (OD) of the stained adherent bacteria were measured at 630 nm wavelength using an enzyme-linked immunosorbent assay (ELISA) plate reader (RT-2100C, Rayto, Shenzhen, China). The experiment included the positive control, *Lactiplantibacillus plantarum* LP 299v (probiotic biofilm-producing strain), and the negative control containing only the culture medium. The biofilm formation assay was performed in triplicate and results were presented as means ± standard deviations. To compensate for background absorbance, OD readings from dyed and fixated non-inoculated wells were averaged and subtracted from test values.

According to Stepanovic et al. [53] the strains were classified into four different categories as follows: OD < ODc, no biofilm producer (0); ODc < OD < 2ODc, weak biofilm producer (+); 2ODc < OD < 4ODc, moderate biofilm producer (++); and 4ODc < OD, strong biofilm producer (+++). The cut-off OD (ODc) was defined as three standard deviations above the mean OD of the negative control wells.

## 3. Results and Discussion

### 3.1. Enumeration, Isolation, and Identification

The total count of viable LAB was higher on M17 agar plates than on MRS or BEA agar plates (Table 1). On MRS and M17 media, the count of viable LAB was lowest on the 0th day, but slightly higher than in goat milk. After the 7th day of ripening, the count started to grow, while after the 21st day, the count began to drop. The total count of enterococci on bile esculin agar (BEA) plates was lowest on the 0th day, but slightly higher than in goat milk. After the 7th day of ripening, the count started to grow, while after the 14th day, the count began to drop. After the 21st day, the count of enterococci stagnated.

The research conducted by Mladenović, Grujović, Kocić-Tanackov, Bulut, Iličić, Degenek and Semedo-Lemsaddek [10] revealed that the total count of aerobic mesophilic bacteria and total enterobacteria peaked on the 14th day of ripening, reaching 5.24 × 10^11^ CFU/g and 1.24 × 10^8^ CFU/g, respectively. Moreover, the authors noted a significant increase in mold growth after 14 days of ripening, leading to the development of mold on the cheese surface by the 28th day. This mold proliferation hindered the isolation of LAB for further analysis after day 28. Mladenović et al. [10] also highlighted that respondents initially reported excellent sensory characteristics of the goat cheese during the first three weeks of ripening. However, after this period, the cheese began to exhibit a bitter taste and a stronger odor, resulting in a decline in the attributed points. Notably, the number of viable LAB observed in this study was comparable to that reported for other homemade cheeses from Serbia up to the 7th day of ripening [7,9,54,55]. Interestingly, none of the aforementioned studies investigated the count of LAB beyond the 28th day of ripening.

After plate counting, presumptive LAB were isolated and submitted to conventional microbiological characterization. All Gram-positive and catalase-negative LAB were further analyzed by biochemical tests (Table 2), API 50CH (Supplementary Table S1), and Microgen Strep ID tests (Supplementary Table S2). For additional confirmation of species allocation, MALDI-TOF mass spectrophotometry was also applied. The results obtained identified the following microorganisms: *Enterococcus faecalis* (49 isolates), *Enterococcus faecium* (13 isolates), *Enterococcus hirae* (9 isolates), *Lactococcus lactis* subsp. *lactis* (89 isolates), *Lacticaseibacillus paracasei* (46 isolates), and *Lactiplantibacillus plantarum* (32 isolates) (Table 3). Representative mass spectra for *L. lactis* subsp. *lactis*, *L. paracasei*, and *L. plantarum* can be seen in Supplementary Figure S1, while representative mass spectra for members from the *Enterococcus* genus can be seen in Supplementary Figure S2.

The findings of our investigation revealed that all isolates harbor the capability to grow within the pH range of 4–7.5. This observation aligns with the documented variations in pH in goat cheese reported by Mladenović, Grujović, Kocić-Tanackov, Bulut, Iličić, Degenek and Semedo-Lemsaddek [10], ranging from 6.55 on day 0 to 4.75 on day 28. Moreover, *Lacticaseibacillus* and *Lactiplantibacillus* exhibited robust growth even at pH 3.5, whereas *Lactococcus lactis* subsp. *lactis* exhibited no growth under similar conditions. The growth ability of *Enterococcus* at pH 3.5 showed strain-dependent characteristics, as delineated in Table 2. Comparable results were reported by Muruzović et al. [56] and Grujović, Mladenović, Žugić-Petrović and Čomić [52] in their investigations of the growth capacity of LAB isolated from raw cow’s cheese (related to different pH conditions).

It is noteworthy that, in addition to *Enterococcus* spp., *Lactiplantibacillus plantarum* exhibited high tolerance to salt, demonstrating growth capability in the presence of 6.5% NaCl, while only a limited number of *Lactococcus lactis* subsp. *lactis* (5/89; 5.62%) displayed tolerance to this salt concentration (see Table 2). Similar findings have been reported by other researchers investigating homemade cheeses [7,55]. The remarkable salt tolerance displayed by *L. plantarum* holds significant implications for bacteria intended for use as starter cultures. In cheese production, for instance, salt plays a pivotal role not only in flavor development but also in preservation and moisture control [57]. Bacteria capable of thriving in high-salt environments, such as *L. plantarum*, contribute to the fermentation process and aid in shaping the desired characteristics of the final product. Their ability to endure salt conditions ensures viability and activity throughout the fermentation process, thereby enhancing the consistency and quality of the product [58]. Furthermore, in settings where salt concentrations fluctuate, salt-tolerant bacteria like *L. plantarum* provide stability and reliability to starter cultures, rendering them indispensable for ensuring consistent and successful fermentation processes across various food production applications.

A significant proportion of the isolates belonging to *Lacticaseibacillus* and *Lactiplantibacillus* genera exhibited the ability to produce Exopolysaccharides (EPSs) (67.39% and 84.37%, respectively). EPS-producing LAB have the capability to modulate the adhesion of probiotics and enteropathogens to the human intestinal mucosa, as demonstrated in previous studies [59]. These cultures play a crucial role in enhancing the sensory attributes of dairy products, given that consumers often prefer smooth and creamy textures [60]. Therefore, EPS production emerges as a pertinent characteristic to evaluate during starter culture selection processes. Terzic-Vidojevic, Tolinacki, Nikolic, Veljovic, Jovanovic, Macej and Topisirovic [55] indicated that numerous lactobacilli from Vlasina goat cheese were EPS producers, corroborating our findings.

Several *Lactococcus lactis* subsp. *lactis* isolates, as well as numerous *Lacticaseibacillus* and *Lactiplantibacillus*, exhibited the capability to metabolize citrate. Such bacteria play a pivotal role in flavor compound synthesis through lactose fermentation and citrate metabolism. Citrate utilization fosters the production of diacetyl, recognized as a primary flavor component in fermented milk products [61]. Nikolic et al. [62] indicated that members of the former *Lactobacillus* genus isolated from goat cheese showed the ability to utilize citrate in high numbers, which aligns with our study. Muruzović, Mladenović, Žugić-Petrović and Čomić [7] also demonstrated that *L. fermentum* isolated from raw cow cheese possesses the capacity to utilize citrate. Furthermore, Muruzović et al. [9] indicated the capacity of *L. lactis* subsp. *lactis* to utilize citrate, which is also confirmed in our study.

### 3.2. Safety Evaluation

The safety of the tested isolates was assessed through evaluations of hemolytic activity and antibiotic sensitivity. Results revealed that *Lactococcus* spp., *Lacticaseibacillus* spp., and *Lactiplantibacillus* spp. did not exhibit α or β hemolysis, indicating an absence of hemolytic activity. Conversely, enterococci displayed α hemolysis and were consequently excluded from further analysis.

Furthermore, the minimum inhibitory concentration (MIC) for five antibiotics (ampicillin, tetracycline, gentamicin, streptomycin, and vancomycin) was determined for the 167 LAB exhibiting no hemolytic activity (refer to Supplementary Table S3). The results were compared against resistance criteria outlined by the European Food Safety Authority (EFSA) for antibiotics of human and veterinary importance in 2018 [63]. Table 4 summarizes the range of MIC values (μg/mL) for the tested isolates.

Among *L. lactis* subsp. *lactis*, the highest proportion exhibited resistance to vancomycin (isolates M-3, M-11, C0-6, C14-3, C14-11, C21-5, C21-11, C21-16, and C28-3), followed by gentamicin (M-11, C0-6, C14-2, C14-18, C21-9, and C28-1), ampicillin (C14-3, and C21-13), tetracycline (C0-13, and C21-5), and streptomycin (C7-5 and C21-19). None of the tested *L. lactis* subsp. *lactis* isolates exhibited resistance to more than three antibiotics but isolates C14-3 and C21-5 were resistant to two of the five antibiotics assessed (see Supplementary Table S3).

Among *L. paracasei*, the majority exhibited resistance to gentamicin (isolates C0-4, C7-4, C14-3, and C14-5) and tetracycline (C0-3 and C7-9). None of the tested *L. paracasei* isolates displayed resistance to streptomycin or ampicillin (see Supplementary Table S3).

Among *L. plantarum*, the highest proportion exhibited resistance to gentamicin (isolates C7-5, C14-2, C14-9, and C21-8) and tetracycline (C14-10). None of the tested *L. plantarum* isolates displayed resistance to ampicillin (see Supplementary Table S3).

Overall, a higher number of isolates demonstrated sensitivity to ampicillin, tetracycline, and streptomycin. Examination results of bacteria isolated from raw cow cheese in southeastern Serbia indicated that all tested LAB were sensitive to ampicillin, tetracycline, gentamicin, and vancomycin [52]. Similarly, Uroić et al. [64] investigated antibiotic sensitivity of LAB isolated from Serbian and Croatian cheeses and found that all isolates were susceptible to antibiotics. However, Leite et al. [65] reported that lactococci isolated from Brazilian kefir were susceptible to tetracycline and ampicillin, with vancomycin susceptibility being the exception. In our study, lactococci exhibited a higher percentage of resistance to vancomycin (10.11%), followed by gentamicin (6.74%).

The absence of antibiotic resistance in starter cultures is crucial for safety reasons because bacteria resistant to antibiotics may transfer their resistance to other bacteria. Out of 167 isolates tested for sensitivity to five different antibiotics, 137 were sensitive to all tested compounds. Consequently, they were selected for further examination as potential starter cultures for cheese production.

### 3.3. Technological Features

Technological features were evaluated using tests including growth in methylene blue, proteolytic activity, assessment of acidification and curd formation ability in both pure and enriched goat milk, diacetyl production, detection of LAB antagonistic potential, and evaluation of biofilm formation ability. Only bacteria deemed safe based on the safety aspect tests (70 *Lactococcus lactis* subsp. *lactis*, 40 *Lacticaseibacillus paracasei*, and 27 *Lactiplantibacillus plantarum*) underwent assessment for technological features.

#### 3.3.1. Growth in Methylene Blue

The methylene blue reduction test operates on the principle that the color imparted to milk by the addition of a dye, such as methylene blue, will gradually fade. This fading occurs due to the removal of oxygen from the milk and the subsequent formation of reducing substances during bacterial metabolism. Bacteria consume oxygen, and the more bacteria present in the milk, the faster the oxygen is depleted, leading to a quicker disappearance of color. Therefore, the time taken for reduction serves as an indicator of the bacterial population in the milk [66].

Consistent with this principle, all tested *Lactococcus* spp., *Lacticaseibacillus* spp., and *Lactiplantibacillus* spp. demonstrated the ability to grow in the presence of methylene blue. This suggests their capacity for growth in milk with significant bacterial counts. Similar findings were reported by Muruzović, Mladenović, Žugić-Petrović and Čomić [7] and Muruzović, Mladenović, Djilas, Stefanović and Čomić [9], who investigated the growth capacity in the presence of methylene blue of LAB isolated from raw cowʹs cheese.

#### 3.3.2. Proteolytic Activity

The production of extracellular proteinases is a crucial characteristic of LAB for curd formation and flavor development. Among the 70 *Lactococcus lactis* subsp. *lactis*, 25 isolates (35.71%) exhibited proteolytic activity. Similarly, among the 40 *Lacticaseibacillus paracasei*, 18 isolates (45%) displayed proteolytic activity. Additionally, out of 27 *Lactiplantibacillus plantarum*, 11 isolates (40.74%) demonstrated proteolytic activity. More detailed information can be found in Supplementary Table S4. It is well documented that these LAB often exhibit superior proteolytic activity compared to other groups of LAB such as enterococci [67,68,69].

The proteolytic activity of LAB potentially utilized as starter cultures is fundamental in cheese production. These enzymes serve a pivotal role in the process of curd formation by enzymatically hydrolyzing milk proteins, particularly casein, thereby contributing to the desired texture and structural integrity of the cheese [67]. Additionally, proteolysis facilitates flavor development during cheese ripening by liberating peptides and amino acids, acting as precursors for flavor compounds, which in turn enhance the sensory attributes of the cheese. Moreover, the impact of proteolytic activity extends to influencing cheese texture, affecting parameters such as firmness, elasticity, and smoothness [70]. Furthermore, lactic acid bacteria proteolytic activity on caseins gives rise to small peptides displaying antimicrobial (both bactericidal and bacteriostatic) activity [71]. Therefore, proper proteolysis also contributes to the shelf life and stability of cheese, thereby ensuring its quality is maintained throughout the storage and distribution processes.

#### 3.3.3. Milk Acidification and Curd Formation

**Figure 1:**
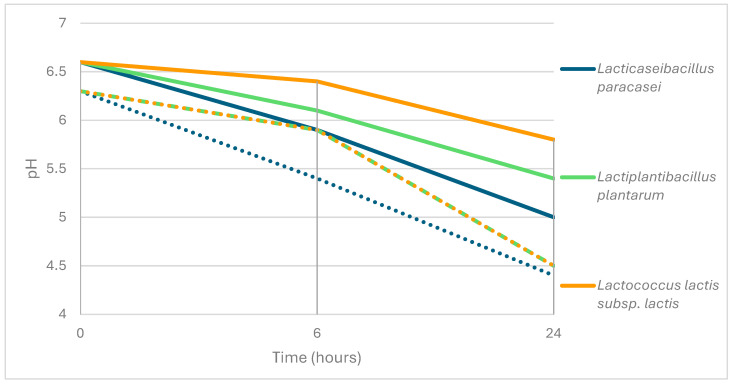
pH variation throughout the assay. Dotted lines represent pH evolution in enriched milk, whereas solid lines represent pure milk.

The activity in pure and enriched goat milk was evaluated for isolates that showed proteolytic activity. The activity of goat milk and cheese isolates was initially limited after 6 h of incubation but improved significantly after 24 h. pH variation is presented in Figure 1.

*Lacticaseibacillus paracasei* exhibited the ability to form curds after 24 h of incubation in both pure and enriched milk. In comparison to pure goat milk (pH 6.6), these isolates demonstrated acidification ability, with a pH of approximately 5.9 at 6 h and 5 at 24 h. In enriched milk (pH 6.3), the acidification ability was enhanced, with a pH of about 5.4 at 6 h and 4.4 at 24 h.

Similarly, *Lactiplantibacillus plantarum* isolates displayed the ability to form curds after 24 h of incubation in pure and enriched milk. These isolates also exhibited acidification ability, with a pH of around 6.1 at 6 h and 5.4 at 24 h in pure goat milk (pH 6.6), and improved acidification in enriched milk (pH 6.3) with a pH of about 5.9 at 6 h and 4.5 at 24 h.

Furthermore, *Lactococcus lactis* subsp. *lactis* demonstrated formed curds after 24 h of incubation in pure and enriched milk. Acidification ability was observed, with a pH of approximately 6.4 at 6 h and 5.8 at 24 h in pure goat milk (pH 6.6), and enhanced acidification in enriched milk (pH 6.3) with a pH of about 6.1 at 6 h and 5.5 at 24 h.

The findings of this study highlight the curd-forming ability and acidification properties of *Lacticaseibacillus paracasei*, *Lactiplantibacillus plantarum*, and *Lactococcus lactis* subsp. *lactis* isolates in both pure and enriched milk. Other studies indicated that LAB from raw cows and goat milk showed acidification activity, as well as the ability of curd formation [7,9,55]. The capacity of these isolates to form curds underscores their potential as starter cultures in cheese production. Moreover, their acidification ability, characterized by a decrease in pH over time, suggests their effectiveness in fermenting milk and creating the acidic environment necessary for cheese production. Importantly, the enhanced acidification observed in enriched milk underscores the influence of milk composition on bacterial activity and highlights the potential for optimizing fermentation conditions to achieve desired product characteristics. According to Mladenović et al. [10], the pH of the investigated goat cheese was below 5 after the 14th day of ripening. This data aligns with the cheese’s higher count of viable LAB (Table 1). Therefore, to enhance food safety and hygiene practices among small-scale dairy producers, it is crucial to use multigrain starters in curd cheese production, as not all mesophilic bacteria can produce sufficient lactic acid to lower the pH below 5.

#### 3.3.4. Diacetyl Production

All LAB that demonstrated proteolytic activity and milk coagulation ability were tested for their ability to produce diacetyl. The results indicated that among 18 *Lacticaseibacillus paracasei*, 11 were able to produce diacetyl, while 9 out of 11 *Lactiplantibacillus plantarum* exhibited diacetyl production (see supplementary Table S4). These findings align with those reported by Nikolic, Terzic-Vidojevic, Jovcic, Begovic, Golic and Topisirovic [62]. However, Terzic-Vidojevic, Tolinacki, Nikolic, Veljovic, Jovanovic, Macej and Topisirovic [55] reported that potential starter cultures (*L. lactis* subsp. *lactis* BGVL2-8 and *L. plantarum* BGVL2a-18) from their study did not demonstrate the ability to produce diacetyl. Additionally, *L. lactis* subsp. *lactis* from our study did not exhibit diacetyl production.

As previously mentioned, diacetyl, a primary flavor component in fermented milk products, is typically generated through citrate utilization by bacteria [61]. Controlled and managed diacetyl production by starter cultures can enhance the flavor and aroma of dairy products. However, it is crucial to note that excessive diacetyl production or inconsistency in its levels may lead to undesirable off-flavors and quality issues [15]. Therefore, the suitability of diacetyl production as a characteristic of starter cultures depends on specific product requirements and consumer preferences.

#### 3.3.5. Biogenic Amine Production

All lactic acid bacteria exhibiting proteolytic activity and milk coagulation capacity underwent assessment for their potential to produce biogenic amines. Results revealed that none exhibited the ability to produce biogenic amines from histidine and tyrosine (refer to Supplementary Table S4), indicating a desirable characteristic for potential use as starter cultures. This aligns with findings from previous studies conducted by Terzic-Vidojevic, Tolinacki, Nikolic, Veljovic, Jovanovic, Macej and Topisirovic [55] and Grujović, Mladenović, Žugić-Petrović and Čomić [52], which investigated LAB from raw goat and cow milk cheese originating from Serbia. Nonetheless, as underscored by Terzic-Vidojevic, Tolinacki, Nikolic, Veljovic, Jovanovic, Macej and Topisirovic [55], before the implementation of selected LAB strains in cheese production, a comprehensive qualitative and quantitative analysis of biogenic amines is imperative.

#### 3.3.6. Antagonistic Potential

In this study, we assessed LAB’s potential to inhibit the growth of indicator bacteria using the agar-well diffusion method. The indicator strains included *E. coli* ATCC 25922, *P. mirabilis* ATCC 12453, *S. aureus* ATCC 25923, *E. coli* G14, and *K. pneumoniae*. Table 5 displays the results, showing the range of inhibition zone diameters for the different genera (considering isolates where the inhibition zone diameters exceeded 6 mm for at least one indicator strain). More detailed information can be found in Supplementary Table S5.

Tested indicator strains exhibited sensitivity to all antibiotics tested (Table 6), except *P. mirabilis* ATCC 12453, which displayed resistance to tetracycline (10 mm).

Other studies have demonstrated the antagonistic potential of non-starter LAB against various bacterial species. For instance, Muruzović, Mladenović, Žugić-Petrović and Čomić [7] reported moderate antagonistic activity of *Lactobacillus* sp. and *Lactococcus* sp. against *Escherichia coli* ATCC 25922, *Proteus mirabilis* ATCC 12453, *Klebsiella oxytoca* KGPMF1, *Klebsiella ornithinolytica* KGPMF8, and *Aeromonas hydrophila*, relative to antibiotics. Fraga et al. [72] demonstrated promising activity of *L. lactis* 8L1A and 8L1B isolated from cheese against bacterial pathogens.

Through comparative analysis with antibiotic inhibition zone diameters against various indicator strains in our investigation, it is evident that certain LAB isolates surpassed the inhibitory activity of antibiotics. Specifically, among the isolates, *L. paracasei* (M-1, C14-1, C14-9, C14-16, C21-1, and C21-2), as well as *L. plantarum*, demonstrated the most potent antagonistic effects against the indicator strains compared to the antibiotics. Other isolates exhibited moderate inhibitory activity against the tested Gram-negative bacteria in comparison to the antibiotics.

This attribute of LAB, potentially utilized as starter cultures, holds significance as bacteria exhibiting antagonistic potential against food-spoilage organisms not only function as starter cultures but also contribute to cheese preservation. Fraga, Schein, Giacaman, Zunino and Techera [72] suggested that LAB strains selected as starters could effectively contribute to cheese preservation and safety.

#### 3.3.7. Biofilm Formation Ability of Tested LAB

When discussing biofilms, the conversation often revolves around the myriad risks they entail, including human diseases, antibiotic resistance, infections, and their resilience to disinfection and cleaning methods [73]. However, it is important to acknowledge that not all biofilms contribute to these negative outcomes across various domains. Certain types of LAB biofilms, for instance, can function as protective barriers against pathogens and their own biofilms [74]. The assessment of LAB isolates’ ability to form biofilms is crucial for their probiotic applications. LAB biofilms are commonly found in food environments, including food processing plants, food products, milk, meat, plants, the gastrointestinal tract, human mucosae, vaginal areas, as well as domestic and industrial settings [74]. LAB biofilms significantly contribute to the establishment and persistence of these bacteria in the cheese-making environment, thereby helping maintain specific organoleptic features of the cheese over time [37]. These biofilms provide a stable and protected environment for LAB, allowing them to adhere to surfaces and persist through cleaning processes [74,75], ensuring their continuous presence in cheese production [37]. The biofilm matrix protects LAB from harsh conditions, facilitates efficient colonization of fresh cheese curds, and ensures the consistent production of metabolic compounds essential for flavor and texture [74,76]. Additionally, LAB biofilms offer a competitive advantage by inhibiting undesirable microorganisms [75,77], leading to consistent fermentation processes and uniform cheese characteristics [37]. This persistence and activity of LAB biofilms are crucial for maintaining the quality, safety, and sensory appeal of the cheese across production cycles.

The biofilm formation results presented in this paper, evaluated using the crystal violet method, revealed that LAB isolates predominantly displayed moderate biofilm-producing capabilities. Understanding the phenotype of LAB biofilms holds promise for gaining novel insights into enhancing their antimicrobial properties. Additionally, there is a burgeoning interest in harnessing LAB biofilms for biocontrol purposes against pathogenic microorganisms, as highlighted by Mgomi, Yang, Cheng and Yang [74]. Table 7 summarizes biofilm formation abilities, ranging from weak to strong biofilm producers.

Among the 25 *L. lactis* subsp. *lactis* isolates with commendable technological features, seven exhibited the ability to form biofilms. Notably, *L. lactis* subsp. *lactis* C0-14 and C21-8 were classified as strong biofilm producers. Similarly, out of the 18 *L. paracasei* possessing favorable technological attributes, eight demonstrated the capacity to form biofilms. Noteworthy are *L. paracasei* M-1 and C7-13, identified as strong biofilm producers.

In the case of the 11 *L. plantarum* characterized by their relevant technological traits, eight displayed biofilm-forming abilities. Notably, *L. plantarum* C7-7, C7-8, and C14-5 were classified as strong biofilm producers.

The utilization of LAB biofilms for biocontrol against pathogenic microorganisms has garnered increasing attention in recent years. Previous studies on LAB isolated from Sokobanja cheese have demonstrated their capacity for biofilm formation [9]. However, the quantity of biomass produced within biofilms typically varies depending on the specific strain, a phenomenon supported by our current investigation, as indicated by Diaz et al. [78]. Pérez Ibarreche et al. [79] further illustrated that *Lactobacillus* spp. capable of forming biofilms exhibited efficacy in controlling the development of *Listeria monocytogenes* on abiotic surfaces. Consistent with these findings, our study confirmed the antagonistic effect of isolates with biofilm-forming abilities. Notably, certain isolates of *L. lactis* subsp. *lactis*, *L. paracasei*, and *L. plantarum* emerged as strong biofilm producers, underscoring their potential for diverse applications within the food industry and beyond.

## 4. Conclusions

In this study, among 238 LAB isolates, 137 were identified as safe based on various evaluation tests, including hemolytic activity and antibiotic sensitivity assays. These selected isolates were further examined for technological properties. Proteolytic activity, acidification activity, and the ability to form curds were observed in 25 *Lactococcus lactis* subsp. *lactis*, 18 *Lacticaseibacillus paracasei*, and 11 *Lactiplantibacillus plantarum*. Among them, 11 isolates of *L. paracasei* and 9 *L. plantarum* exhibited diacetyl production. None of the tested isolates were found to produce histamine or tyramine. The antagonistic effect of the investigated LAB was demonstrated against five indicator strains, with some isolates surpassing the inhibitory activity of widely used antibiotics. Notably, certain *L. paracasei* and *L. plantarum* exhibited potent antagonistic effects against the indicator strains. Additionally, certain LAB were identified as strong biofilm producers, particularly *L. lactis* subsp. *lactis* isolates C0-14 and C21-8, *L. paracasei* isolates M-1 and C7-13, and *L. plantarum* isolates C7-7, C7-8, and C14-5.

Based on the evaluation of technological properties, *L. paracasei* and *L. plantarum* species showed promising characteristics, making them potential candidate strains for inclusion in starter cultures for goat’s milk cheese production. Notable among *L. paracasei* M-1 isolate exhibited a wide range of desirable properties, including proteolytic activity, acidification ability, curd formation, antimicrobial potential, diacetyl production, strong biofilm formation, and absence of biogenic amine production. Similarly, among *L. plantarum* isolates, C7-7, C7-8, and C14-5 stood out as promising strains with similar advantageous attributes. Among *L. lactis* subsp. *lactis*, C0-14, and C21-8 emerged as promising strains with notable technological properties, making them suitable candidates as starter or adjunct cultures for cheese production.

## Figures and Tables

**Table 1:** Total number of viable LAB in goat milk and cheese samples.

Origin	Day of Ripening	MRS Agar	M17 Agar	BEA Agar
Goat milk ^a^	-	(1.9 ± 0.3) × 10^3^	(2 ± 0.4) × 10^3^	(9.6 ± 0.1) × 10^2^
Goat cheese ^b^	0	(3.2 ± 0.1) × 10^4^	(7.6 ± 0.3) × 10^4^	(5.6 ± 0.2) × 10^3^
	7th	(2.04 ± 0.8) × 10^7^	(3.8 ± 0.7) × 10^9^	(7 ± 0.2) × 10^8^
	14th	(1.5 ± 0.7) × 10^10^	(1.13 ± 0.6) × 10^12^	(9 ± 0.2) × 10^9^
	21st	(7.2 ± 0.2) × 10^9^	(2.56 ± 0.5) × 10^11^	(4 ± 0.1) × 10^6^
	28th	(2.4 ± 0.5) × 10^7^	(2.1 ± 0.1) × 10^6^	(2.6 ± 0.1) × 10^6^

^a^ CFU/mL of milk, average values of three independent experiments; ^b^ CFU/g of cheese, average values of three independent experiments.

**Table 2:** Physiological and technological characteristics of isolated LAB.

Species		*E. faecalis*	*E. faecium*	*E. hirae*	*Lactococcus lactis* Subsp. *lactis*	*L. paracasei*	*L. plantarum*	*L.* *plantarum* LP 299v	*E. faecalis* ATCC 29211
Biochemical characteristics	Morphology	cocci	cocci	cocci	cocci	rods	rods	rods	cocci
	Production of exopolysaccharides	-	-	-	-	+- (31)	+- (27)	+	-
	Growth at 15 °C	+	+	+	+	+	+	+	+
	Growth at 45 °C	+	+	+	-	-	-	-	+
	Growth at 4% of NaCl	+	+	+	+	+	+	+	+
	Growth at 6.5% of NaCl	+	+	+	+- (5)	-	+	+	+
	Growth at 8% of NaCl	+	+	+	-	-	-	-	+
	Growth at pH 3.5	+- (8)	+- (3)	-	-	+	+	+	-
	Growth at pH 4	+	+	+	+	+	+	+	+
	Growth at pH 6.5	+	+	+	+	+	+	+	+
	Growth at pH 7.5	+	+	+	+	+	+	+	+
	Hydrolysis of arginine	+	+	+	-	-	-	-	+
	Hydrolysis of esculin	+	+	+	+	-	+	+	+
	Hippurate hydrolysis	+	+	+	n.d.	n.d.	n.d.	n.d.	+
	Black zone on bile esculin agar	+	+	+	-	-	-	-	-
	Utilization of citrate	-	-	-	+- (11)	+- (31)	+- (22)	-	-
	Production of CO_2_	-	-	-	-	-	-	-	-
MALDI-TOF score		2.26 to 2.45	2.21 to 2.31	2.03 to 2.21	2.14 to 2.16	2.10 to 2.23	2.06 to 2.12	n.d.	n.d.

“+” Positive reaction; “-” negative reaction; “+-” strain-dependent reaction; n.d., not determined; in parentheses is the number of isolates with a positive reaction.

**Table 3:** Distribution of LAB species in goat milk and cheese during ripening.

Origin	Day of Isolation	Species						Total Number of Isolates
		*E. faecalis*	*E. faecium*	*E. hirae*	*Lactococcus lactis* Subsp. *lactis*	*L. paracasei*	*L. plantarum*	
Goat milk	-	8	n.d.	n.d.	12	3	/	23
Goat cheese	0	11	n.d.	v	14	5	3	33
	7th	5	n.d.	n.d.	14	13	8	40
	14th	4	3		18	16	11	52
	21st	6	6	2	22	8	8	52
	28th	15	4	7	9	1	2	38
Total number of isolates		49	13	9	89	46	32	238

**Table 4:** LAB antibiotic sensitivity.

Species	Ampicillin	Tetracycline	Gentamicin	Streptomycin	Vancomycin
*L. lactis* subsp. *lactis*	0.097–3.12	0.097–4.68	0.39–50	0.78–50	0.097–6.24
*L. paracasei*	0.195–3.12	0.195–6.24	1.56–50	1.56–50	n.r.
*L. plantarum*	0.097–1.56	1.56–37.5	1.56–25	n.r.	n.r.
*L. plantarum LP 299v*	6.24	0.125	n.d.	n.r.	n.r.

Values represent minimal inhibitory concentrations (MICs) given in µg/mL; n.r. not required according to EFSA; n.d. not determinated.

**Table 5:** Antagonistic potential against selected indicator strains.

Species	Indicator Strains				
	*S. aureus* ATCC 25923	*P. mirabilis* ATCC 12453	*E. coli* ATCC 25922	*E. coli* G14	*K. pneumoniae*
	ZI* (mm)	ZI* (mm)	ZI* (mm)	ZI* (mm)	ZI* (mm)
*L. lactis* subsp. *lactis*	8–16	0–12	0–8	8–12	0–12
*L. paracasei*	6–18	6–16	6–14	0–12	0–12
*L. plantarum*	12–18	8–14	10–16	0–12	0–10

ZI*, zone of growth inhibition given in mm (millimeter).

**Table 6:** Antibiotic susceptibility of indicator strains.

Antibiotic	*S. aureus* ATCC 25923	*P. mirabilis* ATCC 12453	*E. coli* ATCC 25922	*E. coli* G14	*K. pneumoniae*
Amoxicillin	24 (S)	24 (S)	16 (S)	20	n.d.
Chloramphenicol	26 (S)	45 (S)	31 (S)	24	22
Tetracycline	28 (S)	10 (R)	22 (S)	20	20

Zone of growth inhibition given in mm (millimeter); S-sensitive; R-resistant.

**Table 7:** Biofilm formation ability.

Species	Isolate	Biofilm Quantification	Classification
*L. lactis* subsp. *lactis*	C0-4	0.05 ± 0.02	+
*L. lactis* subsp. *lactis*	C0-14	0.09 ± 0.05	+++
*L. lactis* subsp. *lactis*	C14-7	0.06 ± 0.03	++
*L. lactis* subsp. *lactis*	C14-13	0.06 ± 0.05	++
*L. lactis* subsp. *lactis*	C21-7	0.07 ± 0.03	++
*L. lactis* subsp. *lactis*	C21-8	0.10 ± 0.02	+++
*L. lactis* subsp. *lactis*	C21-21	0.08 ± 0.03	++
*L. paracasei*	M-1	0.13 ± 0.01	+++
*L. paracasei*	M-3	0.05 ± 0.01	++
*L. paracasei*	C0-1	0.08 ± 0.02	++
*L. paracasei*	C7-6	0.02 ± 0.01	+
*L. paracasei*	C7-11	0.04 ± 0.01	+
*L. paracasei*	C7-13	0.09 ± 0.02	+++
*L. paracasei*	C14-1	0.05 ± 0.03	+
*L. paracasei*	C14-9	0.03 ± 0.01	+
*L. plantarum*	C0-2	0.08 ± 0.03	++
*L. plantarum*	C0-3	0.07 ± 0.02	++
*L. plantarum*	C7-7	0.11 ± 0.03	+++
*L. plantarum*	C7-8	0.12 ± 0.03	+++
*L. plantarum*	C14-1	0.06 ± 0.02	++
*L. plantarum*	C14-3	0.08 ± 0.02	++
*L. plantarum*	C14-5	0.10 ± 0.02	+++
*L. plantarum*	C14-6	0.04 ± 0.01	+
*L. plantarum*	LP 299v	0.14 ± 0.02	+++

The results are presented as mean value ± SD from three independent experiments; weak biofilm producer (+); moderate biofilm producer (++); and strong biofilm producer (+++).

## Data Availability

The original contributions presented in the study are included in the article/supplementary material, further inquiries can be directed to the corresponding author/s.

## Supplementary Materials

The following supporting information can be downloaded at: https://www.mdpi.com/article/10.3390/foods13132065/s1, Figure S1: Mass spectra of (A) *Lactiplantibaacillus plantarum*; (B) *Lacticaseibacillus paracasei*; (C) *Lactococcus lactis* subsp. *Lactis*; Figure S2: Mass spectra of (D) *Enterococcus faecalis*; (E) *Enterococcus faecium*; (F) *Enterococcus hirae*; Table S1: API 50 CHL results of the different isolates; Table S2: The sugar fermentation ability of Enterococcus spp. using the Microgen Strep ID test; Table S3: Antibiotic sensitivity of isolated lactic acid bacteria; Table S4: Technological properties of selected LAB; Table S5: Antagonistic potential of isolated LAB.
