# Supplementary material for: Unveiling the Potential of Lactic Acid Bacteria from Serbian Goat Cheese
Source: Foods. 2024 Jun 28;13(13):2065. doi: 10.3390/foods13132065 (PMC11241559; doi:10.3390/foods13132065)
Supplement: Supplementary file 1 [file foods-13-02065-s001.zip › foods-3070518-supplementary.pdf]

Supplementary Table S1. API 50 CHL results

Test/species	*L. plantarum*	*L. paracasei*	*L. lactis* subsp. *lactis*	*L. plantarum* LP 299v
Glycerol	-	-	-	-
Erythritol	-	-	-	-
D-arabinose	-	-	-	-
L-arabinose	-	+-	+-	+
Ribose	-	+	+	-
D-xylose	-	+-	-	-
L-xylose	+-	-	-	-
Adonitol	+-	-	-	-
ß methyl-D-xyloside	+	-	-	-
Galactose	+-	+	+	+
Glucose	+-	+	+	+
Fructose	+-	+	+-	+
Mannose	+-	+	+	+
Sorbose	-	+-	-	-
Rhamnose	+-	-	-	-
Dulcitol	-	-	-	-
Inositol	-	-	-	-
Mannitol	+	+-	-	+
Sorbitol	+-	+-	-	-
α-methyl-D-mannoside	+-	-	-	-
α-methyl-D-glucoside	+-	-	-	-
N-acetyl-glucosamine	+	+-	+	+
Amygdalin	+	+	+-	+
Arbutin	+	+	+-	+
Esculin	+	-	+	+
Salicin	+	+-	+	+
Cellobiose	+	+	+-	+
Maltose	+	+	+	+
Lactose	+	+	+-	+
Melibiose	-	+-	-	-
Saccharose	+	+	-	+
Trehalose	+	+	+	+
Inulin	-	-	-	-
Melezitose	+	+	-	+
Raffinose	-	+-	-	-
Starch	-	-	+-	-
Glycogen	-	-	-	-
Xylitol	-	-	-	-
ß-gentiobiose	+-	+	+-	+
D-turanose	-	+	-	-
D-lyxose	-	-	-	-
D-tagatose	-	+-	-	-
D-fucose	-	-	-	-
L-fucose	-	-	-	-
D-arabitol	-	-	-	-
L-arabitol	-	-	-	-
Gluconate	-	-	-	-
2-keto-gluconate	-	-	-	-
5-keto-gluconate	-	-	-	-

″+″-positive reaction; ″-″-negative reaction; ″+-″ - isolate depending reaction

Supplementary Table S2. The sugar fermentation ability of *Enterococcus* spp. using the Microgen Strep ID test

Test/Species	*E. faecium*	*E. faecalis*	*E. hirae*	*E. faecalis* ATCC 29211
Melibiosis	+-	+-	+	-
Sorbitol	-	+-	-	+
Inulin	-	-	-	-
Lactose	+-	+	+	+
Arabitol	-	-	-	-
Ribose	+-	+	+	+

″+″-positive reaction; ″-″-negative reaction; ″+-″ - isolate depending reaction

Supplementary Table S3. Antibiotic sensitivity of lactic acid bacteria

**Species**	Isolate	Ampicillin	Tetracycline	Gentamicin	Streptomycin	Vancomycin
L. lactis subsp. *lactis*	M-1	0.78	2.34	12.5	9.375	1.56
L. lactis subsp. *lactis*	M-2	0.097	0.39	3.125	1.56	0.78
L. lactis subsp. *lactis*	M-3	0.39	0.195	6.25	25	4.68
L. lactis subsp. *lactis*	M-4	1.56	3.12	25	12.5	0.78
L. lactis subsp. *lactis*	M-5	0.195	1.56	2.34	9.375	3.12
L. lactis subsp. *lactis*	M-6	0.39	0.195	1.56	3.125	0.195
L. lactis subsp. *lactis*	M-7	0.39	0.097	3.125	12.5	1.56
L. lactis subsp. *lactis*	M-8	0.78	0.78	18.75	9.375	3.12
L. lactis subsp. *lactis*	M-9	0.195	3.12	12.5	25	0.78
L. lactis subsp. *lactis*	M-10	1.56	1.17	1.56	0.78	0.39
L. lactis subsp. *lactis*	M-11	1.56	3.12	50	6.25	4.68
L. lactis subsp. *lactis*	M-12	0.195	2.34	3.125	1.56	0.78
L. lactis subsp. *lactis*	C0-1	0.39	3.12	18.75	25	3.12
L. lactis subsp. *lactis*	C0-2	0.78	0.39	2.34	6.25	1.56
L. lactis subsp. *lactis*	C0-3	0.097	1.56	6.25	9.375	3.12
L. lactis subsp. *lactis*	C0-4	1.56	2.5	12.5	12.5	3.125
L. lactis subsp. *lactis*	C0-4	0.195	0.78	25	1.56	0.097
L. lactis subsp. *lactis*	C0-5	0.39	0.78	1.56	6.25	0.78
L. lactis subsp. *lactis*	C0-6	0.097	0.15	37.5	25	4.68
L. lactis subsp. *lactis*	C0-7	1.56	3.12	12.5	0.78	0.195
L. lactis subsp. *lactis*	C0-8	1.56	0.39	25	9.375	0.78
L. lactis subsp. *lactis*	C0-9	0.78	2.34	3.125	1.56	0.39
L. lactis subsp. *lactis*	C0-10	0.195	1.56	18.75	12.5	0.78
L. lactis subsp. *lactis*	C0-11	0.195	0.097	6.25	3.125	1.56
L. lactis subsp. *lactis*	C0-12	0.39	2.34	1.56	6.25	1.56
L. lactis subsp. *lactis*	C0-13	0.097	4.68	25	6.25	3.12
L. lactis subsp. *lactis*	C0-14	0.195	3.125	12.5	9.375	3.125
L. lactis subsp. *lactis*	C7-1	0.78	2.34	6.25	3.125	1.56
L. lactis subsp. *lactis*	C7-2	0.39	1.56	18.75	12.5	0.78
L. lactis subsp. *lactis*	C7-3	1.56	0.15	3.125	0.78	0.78
L. lactis subsp. *lactis*	C7-4	0.195	1.56	2.34	6.25	0.39
L. lactis subsp. *lactis*	C7-5	0.39	0.78	25	37.5	1.56
L. lactis subsp. *lactis*	C7-6	1.56	0.39	0.78	3.125	0.195
L. lactis subsp. *lactis*	C7-7	0.097	0.097	6.25	1.56	0.78
L. lactis subsp. *lactis*	C7-8	0.097	1.17	0.39	6.25	1.56
L. lactis subsp. *lactis*	C7-9	1.56	3.12	12.5	25	0.78
L. lactis subsp. *lactis*	C7-10	1.56	0.78	3.125	0.78	2.34
L. lactis subsp. *lactis*	C7-11	0.195	3.12	2.34	12.5	3.12
L. lactis subsp. *lactis*	C7-12	0.39	1.56	6.25	9.375	0.78

L. lactis subsp. *lactis*	C7-13	0.78	0.39	1.56	3.125	0.195
L. lactis subsp. *lactis*	C7-14	0.78	2.34	18.75	6.25	0.78
L. lactis subsp. *lactis*	C14-1	0.195	1.56	0.78	1.56	0.097
L. lactis subsp. *lactis*	C14-2	0.39	0.195	37.5	25	2.34
L. lactis subsp. *lactis*	C14-3	3.12	3.12	25	12.5	4.68
L. lactis subsp. *lactis*	C14-4	1.56	0.78	6.25	18.75	1.56
L. lactis subsp. *lactis*	C14-5	0.097	0.195	3.125	6.25	0.78
L. lactis subsp. *lactis*	C14-6	0.78	2.34	1.56	6.25	0.195
L. lactis subsp. *lactis*	C14-7	1.56	1.56	18.75	12.5	1.56
L. lactis subsp. *lactis*	C14-8	0.195	2.34	12.5	9.375	3.12
L. lactis subsp. *lactis*	C14-9	0.097	1.56	2.34	0.78	1.56
L. lactis subsp. *lactis*	C14-10	0.78	0.195	3.125	1.56	0.195
L. lactis subsp. *lactis*	C14-11	1.56	3.125	6.25	3.125	6.24
L. lactis subsp. *lactis*	C14-12	0.39	0.195	0.78	6.25	1.56
L. lactis subsp. *lactis*	C14-13	0.097	1.56	6.25	12.5	2.5
L. lactis subsp. *lactis*	C14-14	0.78	1.56	2.34	25	3.12
L. lactis subsp. *lactis*	C14-15	1.56	0.78	1.56	3.12	0.39
L. lactis subsp. *lactis*	C14-16	0.39	1.56	12.5	6.25	0.195
L. lactis subsp. *lactis*	C14-17	0.097	0.39	0.78	1.56	3.12
L. lactis subsp. *lactis*	C14-18	1.56	3.125	37.5	12.5	0.39
L. lactis subsp. *lactis*	C12-1	0.78	1.56	6.25	3.125	0.097
L. lactis subsp. *lactis*	C21-2	0.78	3.125	0.39	18.75	1.56
L. lactis subsp. *lactis*	C21-3	1.56	0.78	3.125	6.25	0.195
L. lactis subsp. *lactis*	C21-4	0.78	0.195	18.75	25	3.12
L. lactis subsp. *lactis*	C21-5	0.195	4.68	25	12.5	6.24
L. lactis subsp. *lactis*	C21-6	0.39	1.56	6.25	3.125	3.12
L. lactis subsp. *lactis*	C21-7	0.195	2.5	25	9.375	1.56
L. lactis subsp. *lactis*	C21-8	0.195	3.125	12.5	6.25	1.56
L. lactis subsp. *lactis*	C21-9	0.78	1.56	50	12.5	2.34
L. lactis subsp. *lactis*	C21-10	0.097	0.78	3.125	25	0.39
L. lactis subsp. *lactis*	C21-11	0.78	3.125	6.25	1.56	4.68
L. lactis subsp. *lactis*	C21-12	1.56	2.34	12.5	6.25	0.195
L. lactis subsp. *lactis*	C21-13	3.12	0.195	1.56	3.125	0.78
L. lactis subsp. *lactis*	C21-14	0.195	0.78	2.34	12.5	0.39
L. lactis subsp. *lactis*	C21-15	0.78	0.097	1.56	3.125	0.39
L. lactis subsp. *lactis*	C21-16	0.097	1.56	12.5	6.25	4.68
L. lactis subsp. *lactis*	C21-17	0.097	0.39	12.5	3.125	0.195
L. lactis subsp. *lactis*	C21-18	0.39	2.34	3.125	1.56	0.78
L. lactis subsp. *lactis*	C21-19	0.39	1.56	25	50	0.78
L. lactis subsp. *lactis*	C21-10	0.78	0.195	3.125	12.5	0.39
L. lactis subsp. *lactis*	C21-21	1.56	3.125	12.5	9.375	1.56
L. lactis subsp. *lactis*	C21-22	0.097	0.39	6.25	0.78	0.78
L. lactis subsp. *lactis*	C28-1	0.39	2.34	37.5	6.25	2.34
L. lactis subsp. *lactis*	C28-2	0.195	0.78	9.375	12.5	0.39
L. lactis subsp. *lactis*	C28-3	0.78	0.39	12.5	25	4.68
L. lactis subsp. *lactis*	C28-4	0.78	1.56	2.34	3.125	0.39
L. lactis subsp. *lactis*	C28-5	1.56	3.125	0.39	0.78	0.195
L. lactis subsp. *lactis*	C28-6	1.56	2.34	6.25	1.56	0.78
L. lactis subsp. *lactis*	C28-7	0.39	1.56	25	12.5	0.78
L. lactis subsp. *lactis*	C28-8	0.78	2.34	0.39	25	0.097
L. lactis subsp. *lactis*	C28-9	1.56	3.125	2.34	6.25	0.78
L. paracasei	M-1	3.125	1.56	25	12.5	n.r.
L. paracasei	M-2	0.195	0.78	3.125	37.5	n.r.

L. paracasei	M-3	1.56	3.125	12.5	50	n.r.
L. paracasei	C0-1	0.195	3.125	12.5	25	n.r.
L. paracasei	C0-2	0.78	2.5	6.25	6.25	n.r.
L. paracasei	C0-3	0.78	4.68	3.125	37.5	n.r.
L. paracasei	C0-4	0.39	3.125	37.5	6.25	n.r.
L. paracasei	C0-5	0.195	3.125	6.25	3.125	n.r.
L. paracasei	C7-1	2.5	1.56	12.5	50	n.r.
L. paracasei	C7-2	0.195	3.125	6.25	6.25	n.r.
L. paracasei	C7-3	3.125	0.39	25	37.5	n.r.
L. paracasei	C7-4	1.56	0.78	50	12.5	n.r.
L. paracasei	C7-5	1.56	2.5	12.5	12.5	n.r.
L. paracasei	C7-6	0.195	3.125	6.25	25	n.r.
L. paracasei	C7-7	1.56	3.125	6.25	1.56	n.r.
L. paracasei	C7-8	0.39	0.195	12.5	6.25	n.r.
L. paracasei	C7-9	0.78	6.24	1.56	3.125	n.r.
L. paracasei	C7-10	0.195	3.125	6.25	3.125	n.r.
L. paracasei	C7-11	1.56	1.56	3.125	12.5	n.r.
L. paracasei	C7-12	0.195	3.125	25	25	n.r.
L. paracasei	C7-13	1.56	2.5	3.125	12.5	n.r.
L. paracasei	C14-1	0.195	2.5	12.5	25	n.r.
L. paracasei	C14-2	0.78	1.56	12.5	6.25	n.r.
L. paracasei	C14-3	2.5	3.12	37.5	6.25	n.r.
L. paracasei	C14-4	1.56	1.56	3.125	1.56	n.r.
L. paracasei	C14-5	0.39	0.78	37.5	25	n.r.
L. paracasei	C14-6	0.195	3.125	12.5	12.5	n.r.
L. paracasei	C14-7	1.56	0.195	12.5	12.5	n.r.
L. paracasei	C14-8	0.78	0.125	3.125	3.125	n.r.
L. paracasei	C14-9	0.195	1.56	6.25	6.25	n.r.
L. paracasei	C14-10	0.39	2.5	3.125	37.5	n.r.
L. paracasei	C14-11	1.56	3.125	1.56	25	n.r.
L. paracasei	C14-12	0.78	2.5	6.25	6.25	n.r.
L. paracasei	C14-13	2.5	3.125	25	12.5	n.r.
L. paracasei	C14-14	0.195	3.125	1.56	6.25	n.r.
L. paracasei	C14-15	1.56	0.195	12.5	25	n.r.
L. paracasei	C14-16	0.39	2.5	6.25	1.56	n.r.
L. paracasei	C21-1	0.195	3.125	25	3.125	n.r.
L. paracasei	C21-2	0.78	3.125	3.125	1.56	n.r.
L. paracasei	C21-3	3.12	0.195	25	12.5	n.r.
L. paracasei	C21-4	2.5	3.12	6.25	25	n.r.
L. paracasei	C21-5	0.195	3.125	12.5	1.56	n.r.
L. paracasei	C21-6	0.39	3.125	12.5	3.125	n.r.
L. paracasei	C21-7	3.12	0.78	25	6.25	n.r.
L. paracasei	C21-8	0.195	1.56	12.5	25	n.r.
L. paracasei	C28-1	0.39	0.25	12.5	25	n.r.
L. plantarum	C0-1	0.78	3.125	3.125	n.r.	n.r.
L. plantarum	C0-2	1.56	6.25	3.125	n.r.	n.r.
L. plantarum	C0-3	0.195	12.5	12.5	n.r.	n.r.
L. plantarum	C7-1	0.78	25	12.5	n.r.	n.r.
L. plantarum	C7-2	0.39	6.25	3.125	n.r.	n.r.
L. plantarum	C7-3	1.56	25	12.5	n.r.	n.r.
L. plantarum	C7-4	1.56	3.125	12.5	n.r.	n.r.
L. plantarum	C7-5	0.097	12.5	25	n.r.	n.r.
L. plantarum	C7-6	0.39	6.25	12.5	n.r.	n.r.

L. plantarum	C7-7	0.195	12.5	3.125	n.r.	n.r.
L. plantarum	C7-8	1.56	3.125	1.56	n.r.	n.r.
L. plantarum	C14-1	1.56	3.125	1.56	n.r.	n.r.
L. plantarum	C14-2	0.097	1.56	25	n.r.	n.r.
L. plantarum	C14-3	1.56	6.25	12.5	n.r.	n.r.
L. plantarum	C14-4	0.78	12.5	3.125	n.r.	n.r.
L. plantarum	C14-5	0.195	6.25	12.5	n.r.	n.r.
L. plantarum	C14-6	1.56	3.125	12.5	n.r.	n.r.
L. plantarum	C14-7	0.097	6.25	3.125	n.r.	n.r.
L. plantarum	C14-8	0.78	6.25	12.5	n.r.	n.r.
L. plantarum	C14-9	1.56	3.125	25	n.r.	n.r.
L. plantarum	C14-10	0.097	37.5	3.125	n.r.	n.r.
L. plantarum	C14-11	0.39	12.5	1.56	n.r.	n.r.
L. plantarum	C21-1	1.56	12.5	1.56	n.r.	n.r.
L. plantarum	C21-2	0.78	6.25	3.125	n.r.	n.r.
L. plantarum	C21-3	0.78	3.125	3.125	n.r.	n.r.
L. plantarum	C21-4	1.56	6.25	12.5	n.r.	n.r.
L. plantarum	C21-5	0.097	12.5	12.5	n.r.	n.r.
L. plantarum	C21-6	1.56	12.5	1.56	n.r.	n.r.
L. plantarum	C21-7	1.56	25	12.5	n.r.	n.r.
L. plantarum	C21-8	0.39	6.25	25	n.r.	n.r.
L. plantarum	C28-1	0.097	3.125	3.125	n.r.	n.r.
L. plantarum	C28-2	0.39	25	12.5	n.r.	n.r.
L. plantarum	LP 299v	6.24	0.125	n.d.	n.r.	n.r.

Values present minimal inhibitory concentration (MIC) given in μg/ml; n.r. not required according to EFSA; n.d. not determined

Supplementary Table S5. LAB antagonistic potential

Species/Antibiotics	Isolate	Indicator strains									
		S. aureus ATCC 25923		*P. mirabilis* ATCC 12453		*E. coli* ATCC 25922		*E. coli* G14		*K. pneumoniae*	
		ZI	A	ZI	A	ZI	A	ZI	A	ZI	A
L. lactis subsp. *lactis*	M-5	16	T	/	/	4	T	12	T	12	T
L. lactis subsp. *lactis*	M-10	16	T	/	/	/	/	8	T	12	T
L. lactis subsp. *lactis*	C0-4	10	T	8	C	8	T	8	T	12	C
L. lactis subsp. *lactis*	C0-14	8	C	8	T	4	T	12	T	8	T
L. lactis subsp. *lactis*	C7-12	8	C	12	C	4	T	12	T	/	/
L. lactis subsp. *lactis*	C14-4	12	T	8	C	/	/	8	T	/	/
L. lactis subsp. *lactis*	C14-7	10	C	10	C	6	T	12	R	10	C
L. lactis subsp. *lactis*	C14-13	12	T	12	C	4	T	10	C	8	C
L. lactis subsp. *lactis*	C14-15	8	C	8	C	/	/	12	T	/	/
L. lactis subsp. *lactis*	C21-7	14	T	10	T	8	T	12	T	8	T
L. lactis subsp. *lactis*	C21-8	16	T	10	T	6	T	10	T	10	T
L. lactis subsp. *lactis*	C21-15	16	T	8	T	/	/	12	T	/	/
L. lactis subsp. *lactis*	C21-21	12	T	12	T	8	T	12	T	8	T
L. lactis subsp. *lactis*	C21-22	16	T	8	T	8	T	8	T	8	T
L. lactis subsp. *lactis*	C28-5	12	T	8	T	/	/	8	T	/	/
L. paracasei	M-1	16	T	10	C	8	C	12	T	10	C
L. paracasei	M-3	8	T	10	C	12	T	8	T	10	T
L. paracasei	C0-1	12	T	14	C	12	T	12	T	12	T
L. paracasei	C0-5	10	T	8	T	8	T	/	/	12	T
L. paracasei	C7-5	6	T	6	T	8	T	/	/	12	T
L. paracasei	C7-6	8	C	6	T	6	T	8	T	10	T
L. paracasei	C7-11	10	T	6	T	6	T	6	T	10	T
L. paracasei	C7-13	6	T	6	C	10	C	8	T	6	T
L. paracasei	C14-1	16	T	8	T	14	T	6	T	6	T
L. paracasei	C14-9	18	T	14	T	10	T	10	T	10	T
L. paracasei	C14-10	14	T	14	T	10	T	8	T	8	T
L. paracasei	C14-11	10	T	12	T	8	C	/	/	/	/
L. paracasei	C14-12	14	T	8	C	8	C	/	/	/	/
L. paracasei	C14-14	12	T	8	C	10	T	6	T	8	T
L. paracasei	C14-16	16	T	16	T	12	T	8	T	10	T
L. paracasei	C21-1	16	T	12	T	12	T	12	T	8	T
L. paracasei	C21-2	14	T	12	T	10	T	6	T	/	/
L. paracasei	C21-5	12	T	12	T	8	C	/	/	/	/
L. plantarum	C0-2	18	T	14	T	14	T	8	T	8	T
L. plantarum	C0-3	16	T	12	T	12	T	10	T	6	T
L. plantarum	C7-7	14	T	12	T	12	T	12	T	6	T
L. plantarum	C7-8	18	T	10	T	16	T	12	T	10	T
L. plantarum	C14-1	16	T	14	T	10	T	10	T	8	T
L. plantarum	C14-3	16	T	16	T	16	T	8	T	6	T
L. plantarum	C14-5	14	T	10	T	12	T	10	T	8	T
L. plantarum	C14-6	12	T	10	T	12	T	6	T	6	T
L. plantarum	C14-8	12	T	8	T	12	T	/	/	/	/
L. plantarum	C21-3	14	T	12	T	10	T	8	T	6	T
L. plantarum	C21-6	16	T	12	T	10	T	12	T	6	T

ZI*, zone of growth inhibition given in mm (millimeter); A*, zone appearance (C, clear zone of inhibition; T, turbid zone of inhibition; /, no zone of inhibition)

Supplementary Table S4. Technological properties of selected LAB

Species	Isolate	Parameters					
		Growth in methilen blue	Proteolytic activity	Milk acidification and curd formation	Diacetyl production	Biogenic amine production	
						histamine	tyramine
L. lactis subsp. *lactis*	M-5	+	+	A (+)<br>C (+)	-	-	-
L. lactis subsp. *lactis*	M-10	+	+	A (+)<br>C (+)	-	-	-
L. lactis subsp. *lactis*	C0-1	+	+	A (+)<br>C (+)	-	-	-
L. lactis subsp. *lactis*	C0-4	+	+	A (+)<br>C (+)	-	-	-
L. lactis subsp. *lactis*	C0-10	+	+	A (+)<br>C (+)	-	-	-
L. lactis subsp. *lactis*	C0-11	+	+	A (+)<br>C (+)	-	-	-
L. lactis subsp. *lactis*	C0-14	+	+	A (+)<br>C (+)	-	-	-
L. lactis subsp. *lactis*	C7-2	+	+	A (+)<br>C (+)	-	-	-
L. lactis subsp. *lactis*	C7-3	+	+	A (+)<br>C (+)	-	-	-
L. lactis subsp. *lactis*	C7-8	+	+	A (+)<br>C (+)	-	-	-
L. lactis subsp. *lactis*	C7-12	+	+	A (+)<br>C (+)	-	-	-
L. lactis subsp. *lactis*	C14-1	+	+	A (+)<br>C (+)	-	-	-
L. lactis subsp. *lactis*	C14-4	+	+	A (+)<br>C (+)	-	-	-
L. lactis subsp. *lactis*	C14-7	+	+	A (+)<br>C (+)	-	-	-
L. lactis subsp. *lactis*	C14-13	+	+	A (+)<br>C (+)	-	-	-
L. lactis subsp. *lactis*	C14-15	+	+	A (+)<br>C (+)	-	-	-
L. lactis subsp. *lactis*	C21-1	+	+	A (+)<br>C (+)	-	-	-

L. lactis subsp. *lactis*	C21-7	+	+	A (+)<br>C (+)	-	-	-
L. lactis subsp. *lactis*	C21-8	+	+	A (+)<br>C (+)	-	-	-
L. lactis subsp. *lactis*	C21-12	+	+	A (+)<br>C (+)	-	-	-
L. lactis subsp. *lactis*	C21-15	+	+	A (+)<br>C (+)	-	-	-
L. lactis subsp. *lactis*	C21-21	+	+	A (+)<br>C (+)	-	-	-
L. lactis subsp. *lactis*	C21-22	+	+	A (+)<br>C (+)	-	-	-
L. lactis subsp. *lactis*	C28-4	+	+	A (+)<br>C (+)	-	-	-
L. lactis subsp. *lactis*	C28-5	+	+	A (+)<br>C (+)	-	-	-
L. paracasei	M-1	+	+	A (+)<br>C (+)	+	-	-
L. paracasei	M-3	+	+	A (+)<br>C (+)	+	-	-
L. paracasei	C0-1	+	+	A (+)<br>C (+)	-	-	-
L. paracasei	C0-5	+	+	A (+)<br>C (+)	+	-	-
L. paracasei	C7-5	+	+	A (+)<br>C (+)	+	-	-
L. paracasei	C7-6	+	+	A (+)<br>C (+)	-	-	-
L. paracasei	C7-11	+	+	A (+)<br>C (+)	+	-	-
L. paracasei	C7-13	+	+	A (+)<br>C (+)	+	-	-
L. paracasei	C14-1	+	+	A (+)<br>C (+)	+	-	-
L. paracasei	C14-9	+	+	A (+)<br>C (+)	+	-	-
L. paracasei	C14-10	+	+	A (+)<br>C (+)	-	-	-
L. paracasei	C14-11	+	+	A (+)	-	-	-

				C (+)			
L. paracasei	C14-12	+	+	A (+)<br>C (+)	+	-	-
L. paracasei	C14-14	+	+	A (+)<br>C (+)	+	-	-
L. paracasei	C14-16	+	+	A (+)<br>C (+)	-	-	-
L. paracasei	C21-1	+	+	A (+)<br>C (+)	+	-	-
L. paracasei	C21-2	+	+	A (+)<br>C (+)	-	-	-
L. paracasei	C21-5	+	+	A (+)<br>C (+)	-	-	-
L. plantarum	C0-2	+	+	A (+)<br>C (+)	+	-	-
L. plantarum	C0-3	+	+	A (+)<br>C (+)	-	-	-
L. plantarum	C7-7	+	+	A (+)<br>C (+)	+	-	-
L. plantarum	C7-8	+	+	A (+)<br>C (+)	+	-	-
L. plantarum	C14-1	+	+	A (+)<br>C (+)	+	-	-
L. plantarum	C14-3	+	+	A (+)<br>C (+)	-	-	-
L. plantarum	C14-5	+	+	A (+)<br>C (+)	+	-	-
L. plantarum	C14-6	+	+	A (+)<br>C (+)	+	-	-
L. plantarum	C14-8	+	+	A (+)<br>C (+)	+	-	-
L. plantarum	C21-3	+	+	A (+)<br>C (+)	+	-	-
L. plantarum	C21-6	+	+	A (+)<br>C (+)	+	-	-

″+″-positive reaction; ″-″-negative reaction; A- acid production; C-curd formation

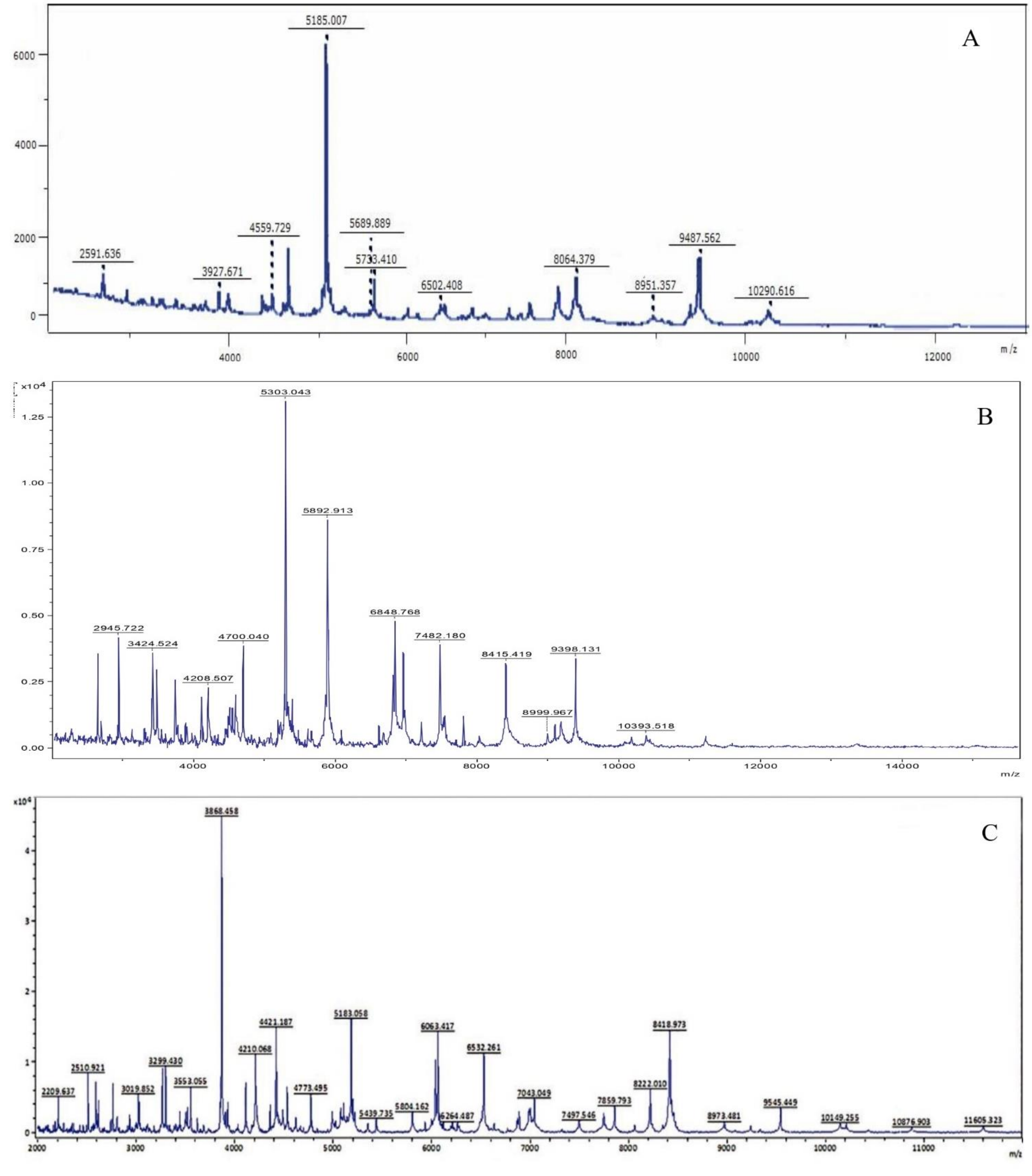

Supplementary Figure 1. Mass spectra of (A) – *Lactiplantibacillus plantarum*; (B) – *Lacticaseibaillus paracasei*; (C) – *Lactococcus lactis* subsp. *lactis*

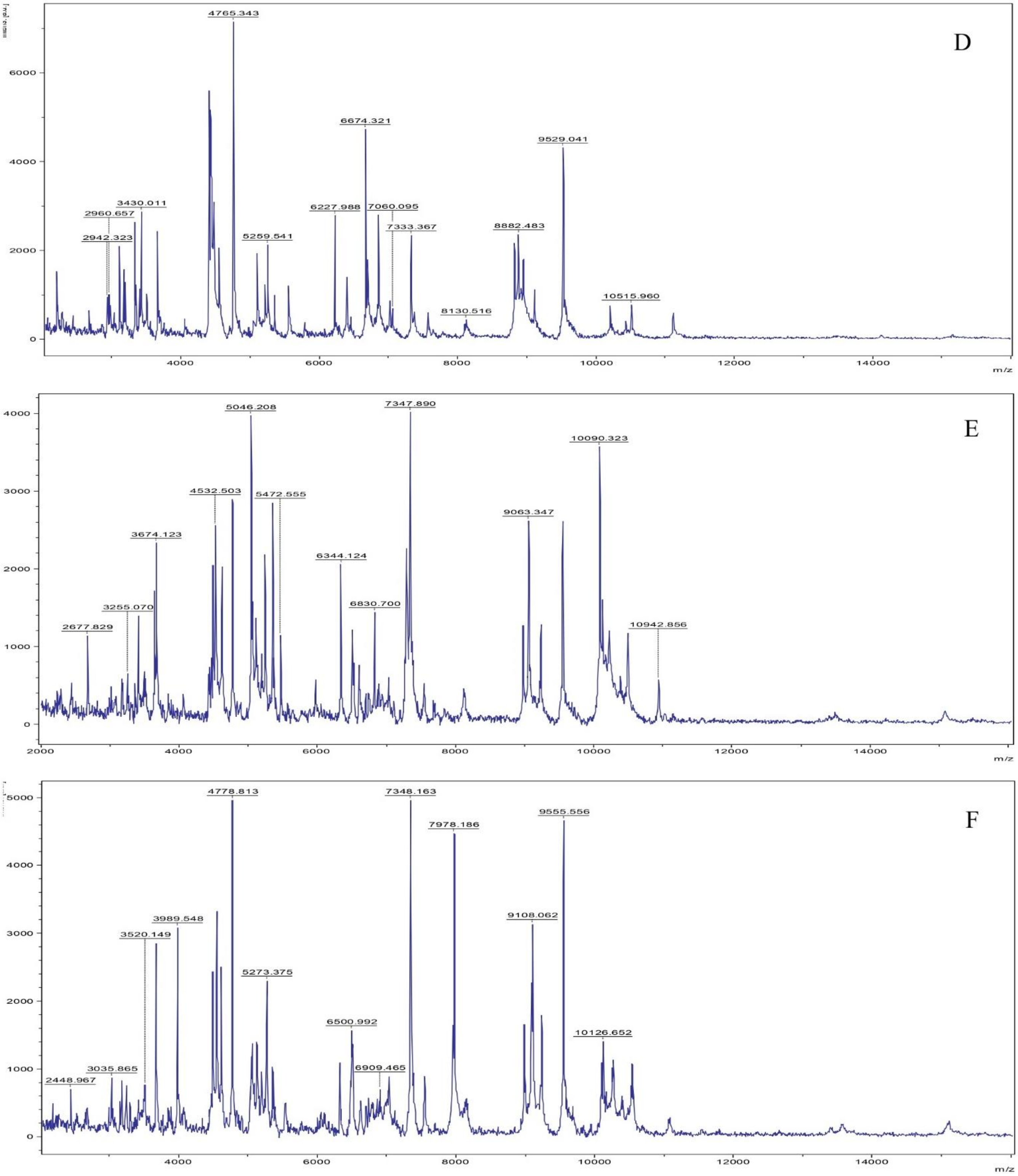

Supplementary Figure 2. Mass spectra of (D) – *Enterococcus faecalis*; (E) – *Enterococcus faecium*; (F) – *Enterococcus hirae*
